# Novel immunotherapeutic strategies for diffuse large B-cell lymphoma: a comprehensive review

**DOI:** 10.3389/fimmu.2026.1704254

**Published:** 2026-03-03

**Authors:** Yixuan Li, Yingying Zhang, Can Zhu, Yimin Han, Xinyun Gao, Juanqing Yue, Ying Wang

**Affiliations:** 1 The Fourth School of Clinical Medicine, Zhejiang Chinese Medical University; Hangzhou First People’s Hospital, Hangzhou, China; 2 School of Pharmacy, Hangzhou Normal University, Hangzhou, China; 3 Affiliated Hangzhou First People’s Hospital, School of Medicine, Westlake University, Hangzhou, China; 4 Clinical Research Center, Affiliated Hangzhou First People’s Hospital, School of Medicine, Westlake University, Hangzhou, China; 5 Laboratory of Medicine, Affiliated Hangzhou First People’s Hospital, School of Medicine, Westlake University, Hangzhou, China; 6 Westlake Laboratory of Life Sciences and Biomedicine, Hangzhou, China

**Keywords:** ADCs, bispecific antibodies, CAR-T cell therapy, diffuse large B-cell lymphoma, immune checkpoint inhibitors, immunotherapy

## Abstract

Diffuse large B-cell lymphoma (DLBCL) represents the most prevalent histological subtype of non-Hodgkin lymphoma, characterized by pronounced clinical and biological heterogeneity. Despite the incorporation of rituximab into the cyclophosphamide, doxorubicin, vincristine, and prednisone (R-CHOP) regimen—achieving durable remission in approximately 60% of patients—30%–40% ultimately experience relapse or develop refractory disease, resulting in unfavorable clinical outcomes. Over the past decade, the advent of novel immunotherapeutic approaches has reshaped the therapeutic paradigm for relapsed/refractory (R/R) DLBCL. Emerging modalities, including monoclonal antibodies, immune checkpoint inhibitors, chimeric antigen receptor T-cell (CAR-T) therapies, antibody–drug conjugates, and bispecific antibodies, have demonstrated encouraging efficacy across multiple clinical settings. Nevertheless, the intrinsic complexity of resistance mechanisms, coupled with the substantial cost and limited accessibility of advanced molecular diagnostics, continues to hinder optimal disease management. This review summarizes the commonly used therapeutic strategies for DLBCL, discusses recent advances, and aims to provide insights for the future development of personalized treatment approaches.

## 1 Introduction

Diffuse large B-cell lymphoma (DLBCL) is the most common subtype of non-Hodgkin lymphoma and also one of the most prevalent malignancies of the lymphoid hematopoietic system, accounting for approximately 30%-40% of all adult cases. It is characterized by high aggressiveness and significant clinical heterogeneity (1, 2). The current standard first-line chemoimmunotherapy regimen is the combination therapy consisting of rituximab, cyclophosphamide, doxorubicin, vincristine, and prednisone (R-CHOP), which can cure approximately 60% of patients (3, 4). However, the survival outcomes for the patients exhibiting R-CHOP resistance are significantly poorer. These patients who fail first-line therapy pose major clinical challenges, highlighting the critical importance of exploring novel treatment strategies to improve the current therapeutic landscape.

Traditionally, DLBCL has been clinically classified into two main subtypes based on cell of origin (COO): the germinal center B-cell (GCB) type and the activated B-cell (ABC) type (5). In 2020, George et al. further categorized DLBCL into seven distinct genetic subtypes based on patient genetic profiles: MCD, BN2, N1, ST2, A53, EZB (MYC+), and EZB (MYC-) (6). These classifications not only reveal the molecular complexity of DLBCL but also provide a theoretical foundation for developing targeted and immunotherapeutic approaches. Recent progress in immunotherapy have offered new therapeutic options for patients with relapsed/refractory (R/R) DLBCL. Multiple studies have demonstrated promising efficacy for emerging immunotherapies, with some patients achieving prolonged disease remission following treatment (7). Common immunotherapeutic strategies include CAR-T cell therapy and immune checkpoint inhibitors. This review focuses on detailing these DLBCL immunotherapeutic strategies, aiming to provide up-to-date references and research directions for the clinical management of DLBCL.

## 2 Novel therapeutic strategies

### 2.1 Monoclonal antibodies

Conventional first-line regimens for DLBCL primarily rely on cytotoxic chemotherapy; however, these treatments are associated with significant toxicities and lack target specificity, highlighting the need for novel therapeutic strategies. CD20 is highly expressed in most B-cell lymphomas and was the first validated therapeutic target. Monoclonal antibodies directed against CD20 can specifically bind to tumor cell surface antigens, induce apoptosis, and significantly improve treatment efficacy with reduced toxicity. Below, we summarize commonly used anti-CD20 monoclonal antibodies.

#### 2.1.1 Rituximab

As a chimeric anti-CD20 IgG1 monoclonal antibody, rituximab was the first widely adopted immunotherapy and has markedly improved patient outcomes. An early multicenter phase II trial reported an overall response rate (ORR) of 37% in 30 patients with first or second relapse of DLBCL treated with rituximab, with some achieving complete remission (CR) (8). Prior to the introduction of rituximab, the standard therapy for DLBCL was the CHOP regimen (cyclophosphamide, doxorubicin, vincristine, and prednisone). In a randomized study of 399 elderly, previously untreated patients, Coiffier et al. (9) compared R-CHOP (CHOP plus rituximab) with CHOP alone. The R-CHOP group demonstrated a CR rate of 76%, significantly higher than the 63% achieved with CHOP, and two-year overall survival (OS) rates of 70% versus 57%, respectively. Similarly, another two-phase trial in elderly, newly diagnosed DLBCL patients reported three-year failure-free survival (FFS) rates of 53% with R-CHOP and 46% with CHOP after a median follow-up of 3.5 years (10). Both studies confirmed the superior efficacy of R-CHOP in prolonging survival compared with CHOP alone.

In addition to these trials, other randomized studies have also demonstrated the clinical benefit of adding rituximab to CHOP (11, 12). Despite the significant survival improvement conferred by R-CHOP, approximately 30% of patients relapse due to resistance, with poor subsequent treatment outcomes. The multicenter phase III CORAL study found that, among 84 patients who failed initial therapy, 58 (69%) had prior rituximab exposure; notably, patients with prior rituximab exposure had inferior response/survival outcomes compared to those without prior rituximab exposure, suggesting that initial rituximab exposure may complicate subsequent treatment and highlight its limited efficacy in certain settings (13). These findings underscore the urgent need to overcome resistance and develop novel therapeutic agents.

#### 2.1.2 Obinutuzumab

Obinutuzumab is a novel glycoengineered type II anti-CD20 monoclonal antibody that induces direct cell death and has demonstrated significant efficacy in follicular lymphoma and chronic lymphocytic leukemia (CLL). To further evaluate its efficacy and safety in DLBCL, the phase II GAUGIN study enrolled 25 patients with relapsed/refractory disease following first-line therapy. Patients were randomized to receive either 1600/800 mg (n = 15) or 400/400 mg (n = 10) dosing regimens. The best overall response rates (ORR) observed were 32% in the 1600/800 mg cohort and 27% in the 400/400 mg cohort (14).

The phase III GOYA trial aimed to compare G-CHOP (obinutuzumab plus CHOP) with R-CHOP (rituximab plus CHOP) in previously untreated patients with advanced DLBCL. A total of 1,418 patients were randomized to receive either regimen. The three-year progression-free survival (PFS) rates were 70% for G-CHOP and 67% for R-CHOP, indicating no significant PFS advantage for obinutuzumab over rituximab (15). In the final analysis of the GOYA study, 1,414 patients (G-CHOP, n = 704; R-CHOP, n = 710) were included, and the five-year PFS rates decreased to 63.8% and 63.6%, respectively, again showing no superiority of G-CHOP. Moreover, the G-CHOP group exhibited a higher incidence of severe adverse events (AEs), including late-onset neutropenia (8.7% vs. 4.9% in the R-CHOP group) (16).

Currently, the use of obinutuzumab in DLBCL remains investigational, and further studies are warranted to define its role and optimize its therapeutic potential.

#### 2.1.3 Tafasitamab

Tafasitamab is an Fc-modified humanized anti-CD19 monoclonal antibody, approved in July 2020 for use in combination with lenalidomide to treat relapsed/refractory diffuse large B-cell lymphoma (R/R DLBCL) (17).

An open-label, randomized, phase 1b clinical trial evaluated the safety and preliminary efficacy of tafasitamab plus lenalidomide combined with R-CHOP (Tafasitamab + Lenalidomide + R-CHOP) as a first-line regimen for treatment-naïve DLBCL. Sixty-six patients were randomized into two groups: Arm T (R-CHOP + tafasitamab) and Arm T/L (R-CHOP + tafasitamab + lenalidomide). ORR and CR rates were 75.8% and 72.7% in Arm T, and 81.8% and 66.7% in Arm T/L, respectively. The 24-month progression-free and overall survival rates were 72.7% and 90.3% (Arm T) and 76.8% and 93.8% (Arm T/L). Promising efficacy signals were observed in both study arms, suggesting potential for this approach in treating DLBCL (18).

To explore the efficacy of tafasitamab combined with other agents, a phase II clinical study evaluated tafasitamab plus lenalidomide in R/R DLBCL patients ineligible for autologous stem cell transplantation. After ≥35 months of follow-up, the ORR was 57.5% (comprising a complete response [CR] rate of 40% and a partial response [PR] rate of 17.5%), with a median progression-free survival (PFS) of 11.6 months and a median overall survival (OS) of 33.5 months (19). As the aforementioned studies were single-arm and lacked a control group, the RE-MIND study was conducted to further assess tafasitamab’s contribution within combination therapy. It compared tafasitamab plus lenalidomide versus lenalidomide monotherapy in DLBCL patients. Results showed significantly higher ORR (67.1% vs. 34.2%) and CR rates (39.5% vs. 13.2%) for the combination therapy compared to monotherapy. This demonstrates that the combination significantly improved response rates and survival outcomes in R/R DLBCL patients, offering a new therapeutic option for this population (20).

A multicenter retrospective study investigating the real-world use of tafasitamab for R/R DLBCL in the US analyzed 181 treated patients. At a median follow-up of 6.5 months, 80% of patients remained alive. The overall response rate (ORR) was 76% and the complete response rate (CRR) was 18% at this timepoint, reflecting a degree of treatment durability and efficacy (21).

### 2.2 Immune checkpoint inhibitors

Immune checkpoints refer to ligand–receptor pairs that exert either inhibitory or stimulatory effects on immune responses (22). Their primary function is to regulate the degree of immune activation during immune responses against pathogens, thereby preventing excessive activation and nonspecific attacks on normal cells, and protecting normal tissues from immune-mediated damage (23).

Immune checkpoint inhibitors work by disrupting coinhibitory signaling pathways involved in tumor immune responses and promoting immune-mediated elimination of tumor cells. They have already demonstrated remarkable efficacy in various malignancies—including melanoma, non-small cell lung cancer, renal cell carcinoma, and bladder cancer and represent a revolutionary milestone in the field of cancer immunotherapy (24–28).

#### 2.2.1 PD-1/PD-L1

##### 2.2.1.1 Mechanism and resistance

Programmed death receptor-1 (PD-1) is a critical immunoinhibitory molecule belonging to the CD28/CTLA-4 superfamily. It binds to its ligands, programmed death-ligand 1 (PD-L1) or programmed death-ligand 2 (PD-L2) (25, 29). PD-L1, a transmembrane glycoprotein of the B7 family, is expressed on activated T and B lymphocytes (30). The PD-1/PD-L1 signaling pathway plays a pivotal role in tumor immune evasion by suppressing T-cell activation and promoting tumor immune tolerance (31). In DLBCL, PD-L1 expression has been associated with poor clinical outcomes and is strongly correlated with the non-GCB subtype (32). Consequently, current research efforts primarily focus on evaluating anti–PD-1 monoclonal antibodies and other checkpoint inhibitors in DLBCL. However, clinical data on the use of anti–PD-1 monotherapy in this setting remain limited.

However, accumulating evidence indicates that the overall clinical efficacy of PD-1/PD-L1 inhibitors in diffuse large B-cell lymphoma (DLBCL) remains limited, largely due to tumor-associated immune escape and the presence of multilayered resistance mechanisms (33–35). The tumor immune microenvironment of DLBCL is highly heterogeneous, and beyond variations in PD-L1 expression, both tumor cells and their surrounding microenvironment can attenuate immune activation following PD-1 blockade through multiple pathways. For instance, enrichment of regulatory T cells (Tregs) and tumor-associated macrophages (TAMs) establishes a persistently immunosuppressive milieu, while upregulation of immunosuppressive cytokines such as interleukin-10 (IL-10) and transforming growth factor-β (TGF-β) further constrains effector T-cell function. In addition, downregulation of antigen-presentation–related molecules, including major histocompatibility complex class I and II (MHC-I/II), together with the development of T-cell functional exhaustion, enables tumor cells to evade immune-mediated clearance despite effective blockade of the PD-1/PD-L1 pathway.

Based on the aforementioned resistance mechanisms, current research is exploring combination immunotherapy strategies to enhance antitumor immune responses. This includes co-administering PD-1/PD-L1 inhibitors with chemotherapy, antibody-drug conjugates (ADCs), or other immunomodulatory agents. Such approaches aim to improve tumor antigen release, reshape the immune microenvironment, and restore effector T-cell function, thereby offering potential to overcome the limitations of monotherapy.

##### 2.2.1.2 Pembrolizumab

As a representative PD-1/PD-L1 pathway inhibitor in DLBCL, the clinical exploration of pembrolizumab in both monotherapy and combination therapy provides important reference for overcoming immune resistance.

Pembrolizumab is a highly selective, fully humanized immunoglobulin G4 (IgG4)-κ monoclonal antibody. It binds to PD-1 on the surface of T cells, thereby blocking the interaction between PD-1 and its ligands PD-L1/PD-L2. This blockade releases T cells from tumor-mediated inhibition (36).

Smith et al. evaluated the safety of pembrolizumab combined with R-CHOP as first-line treatment in treatment-naïve DLBCL patients. Among 29 evaluable patients, the overall response rate (ORR) and complete response (CR) rate were 93% and 83%, respectively. The 18-month progression-free survival (PFS) rate was 82%, and the overall survival (OS) rate was 97%. The results suggest PD-L1 expression may serve as a biomarker to identify DLBCL patients likely to benefit from PD-1 blockade as part of initial therapy (37).

Beyond DLBCL, pembrolizumab is frequently used for relapsed or refractory primary mediastinal large B-cell lymphoma (rrPMBCL). In the multi-cohort phase 1b KEYNOTE-013 trial, 17 relapsed patients were included in the efficacy analysis, achieving an ORR of 41% (7/17). Among 16 patients assessed radiographically, 13 showed reduction in target lesions, indicating pembrolizumab-mediated PD-1 blockade has manageable safety and antitumor activity (38). Another study, KEYNOTE-170, enrolled 53 patients with rrPMBCL who had received at least two prior lines of therapy. The ORR was 45% (CR rate: 13%), with a median PFS of 5.5 months and a 12-month PFS rate of 38%. No treatment-related deaths occurred. These findings further confirm pembrolizumab induces high response rates and durable antitumor activity in rrPMBCL patients, alongside manageable safety. This outcome supported the FDA approval of pembrolizumab for R/R PMBCL following two or more prior lines of therapy (39). Currently, pembrolizumab has become an important treatment option for patients with R/R PMBCL.

##### 2.2.1.3 Nivolumab

Nivolumab is a fully human IgG4 monoclonal antibody targeting PD-1 that blocks PD-1–mediated signaling and restores antitumor immune responses (40).

In a phase II, open-label study, the efficacy and safety of nivolumab were evaluated in patients with relapsed/refractory (R/R) DLBCL who were either ineligible for autologous stem cell transplantation (ASCT) or had relapsed after ASCT. A total of 121 patients were enrolled, including 87 in the post-ASCT failure cohort and 34 in the transplant-ineligible cohort. Patients received four and three doses of nivolumab, respectively. The results showed that the ORR was 10% in the post-ASCT cohort, with a median progression-free survival (PFS) of 1.9 months and a median overall survival (OS) of 12 months. In contrast, the transplant-ineligible cohort demonstrated an ORR of 3%, a median PFS of 1.4 months, and a median OS of 5.8 months. Grade 3–4 adverse events (AEs) occurred in 24% of patients, with neutropenia (4%), thrombocytopenia (3%), and elevated lipase (3%) being the most common toxicities. These findings indicate that, although nivolumab exhibits an acceptable safety profile in this population, its clinical activity as monotherapy remains limited (41). Nevertheless, studies investigating combination strategies with nivolumab are ongoing.

##### 2.2.1.4 Atezolizumab

Atezolizumab is a humanized monoclonal antibody targeting PD-L1. By blocking the interaction between PD-L1 on tumor cells and PD-1 receptors on T cells, it reverses PD-1/PD-L1–mediated immune evasion and restores T-cell–mediated cytotoxicity against cancer cells (42).

An open-label, multicenter phase I/II clinical trial evaluated the efficacy of R-CHOP combined with atezolizumab in previously untreated patients with advanced DLBCL. Patients received R-CHOP–atezolizumab induction therapy for eight cycles. Among 42 enrolled patients, 40 were evaluable for response, yielding an overall response rate (ORR) of 87.5%, including 31 complete responses (CR; 77.5%) and 4 partial responses (PR; 10%). The 6-month overall survival (OS) and progression-free survival (PFS) rates were 100% and 97.4%, respectively, while the 24-month OS and PFS rates were 86.4% and 74.9%. All patients experienced at least one adverse event (AE), with neutropenia (52.4%), constipation (42.9%), and fatigue (40.5%) being the most common. These results demonstrate that the combination therapy achieves high CR rates and favorable survival outcomes, offering a promising treatment option for high-risk DLBCL patients (43).

In addition to combination therapy, a phase II open-label trial investigated atezolizumab as consolidation therapy in DLBCL patients who achieved complete metabolic remission (CMR) after R-CHOP. A total of 109 patients were enrolled, and the 2-year disease-free survival (DFS) rate was 87.9% compared with 79% in historical controls, while the OS rate reached 96.3% versus 87% in the control cohort. These findings suggest that atezolizumab consolidation may improve long-term survival and provide a new avenue for incorporating checkpoint inhibitors into DLBCL treatment strategies (44).

#### 2.2.2 CTLA-4

Cytotoxic T-lymphocyte–associated protein 4 (CTLA-4) is a key inhibitory immune checkpoint molecule that is primarily expressed on activated T cells and regulatory T cells. CTLA-4 shares structural similarity with the costimulatory receptor CD28 but exhibits a higher affinity for the ligands CD80 and CD86 on antigen-presenting cells, thereby competitively inhibiting CD28-mediated costimulatory signaling and negatively regulating early T-cell activation (45, 46).

Clinically, the anti–CTLA-4 monoclonal antibody ipilimumab has been explored in early studies in DLBCL. A phase Ib/II study (NCT03305445) reported long-term follow-up results of a strategy combining nivolumab and ipilimumab in patients with relapsed or refractory DLBCL. Among six heavily pretreated patients, an ORR of 50% was observed (CR rate 16.6%, PR rate 33.3%), with two patients achieving durable CRs lasting more than 80 months. Despite the limited sample size and a relatively high incidence of treatment-related adverse events, these findings suggest that CTLA-4 blockade, when applied in a specific immunological context, may enhance T-cell function and induce durable antitumor responses, providing preliminary clinical support for CTLA-4–based combination immunotherapy strategies in DLBCL (47).

#### 2.2.3 LAG-3

Lymphocyte activation gene-3 (LAG-3) is an important inhibitory immune checkpoint molecule that suppresses immune cell proliferation, activation, and cytokine production upon ligand engagement, thereby promoting T-cell exhaustion and facilitating tumor immune evasion. LAG-3 has been recognized as a promising target for cancer immunotherapy due to its frequent co-expression with other inhibitory receptors and its role in maintaining an immunosuppressive tumor microenvironment (48).

In a phase I open-label study, the efficacy of MGD013, a bispecific DART^®^ molecule simultaneously targeting PD-1 and LAG-3, was evaluated in patients with R/R DLBCL. A total of 17 patients were enrolled, and the study reported an ORR of 36.4%, including one complete response (CR, 9.1%) and three partial responses (PR, 27.3%). Safety analyses indicated that the regimen was generally well tolerated, with treatment-related adverse events occurring in 64.7% of patients; only one patient experienced a grade 3 pneumonia, and no cases of tumor lysis syndrome were observed. Overall, despite the limited sample size, these findings suggest that dual blockade of the PD-1 and LAG-3 pathways demonstrates preliminary antitumor activity in R/R DLBCL and provides early clinical evidence supporting further investigation of LAG-3–targeted therapeutic strategies in this disease (49).

### 2.3 CAR-T therapy

**Figure 1:**
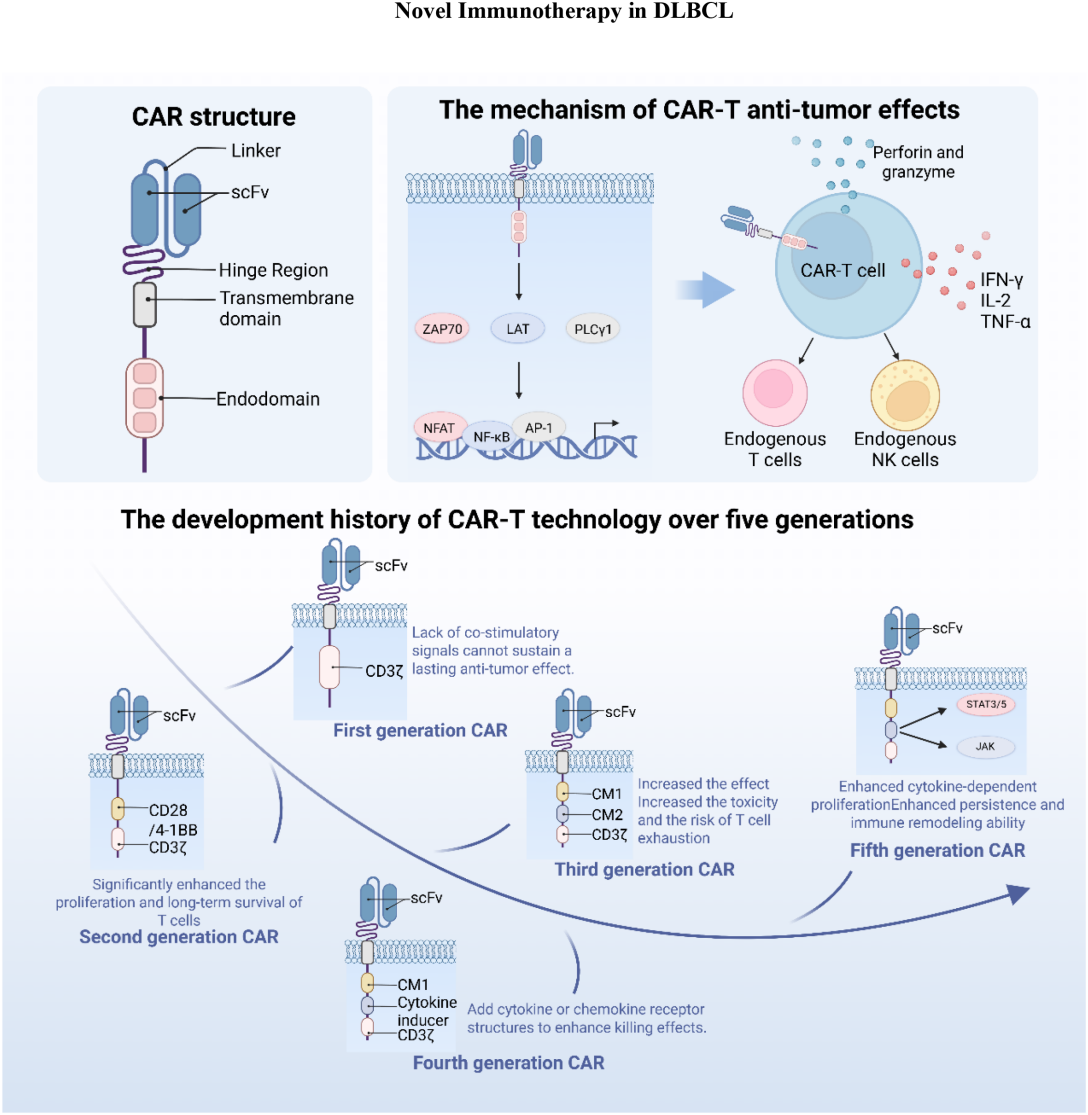
Structural characteristics, signaling mechanisms, and generational evolution of CAR-T cells.

In recent years, with the rapid advancement of tumor immunotherapy, chimeric antigen receptor T-cell (CAR-T) therapy has achieved remarkable success in selectively targeting and eliminating malignant cells (50). As a novel and promising therapeutic strategy, CAR-T therapy has attracted considerable attention in the clinical management of DLBCL, offering a new treatment option for affected patients (51). **Figure 1** illustrates the structural features, signaling mechanisms, and generational evolution of CAR-T cells, providing a framework for the detailed discussion below.

CARs are synthetic receptors designed to redirect T cells toward tumor-specific antigens. They enable T cells to specifically recognize and bind antigens expressed on tumor cell surfaces, leading to T-cell expansion, activation, cytokine release, and robust antitumor responses (52, 53). Structurally, CARs consist of an extracellular antigen-recognition domain and an intracellular signaling domain. The extracellular domain typically comprises a single-chain variable fragment (scFv) derived from antibodies, which is anchored to the cell via a hinge or transmembrane region and linked to intracellular signaling elements, most commonly CD3ζ, to mediate antigen recognition (54, 55). The intracellular domain further promotes T-cell activation and cytotoxicity against tumor cells (56).

CAR-T therapy has now progressed through five generations of development. First-generation CAR-T cells incorporated only the scFv and CD3ζ domains but lacked costimulatory signals, resulting in suboptimal T-cell activation. To address this limitation, second-generation CAR-T cells integrated costimulatory domains, such as CD28 or OX40, in combination with CD3ζ to enhance T-cell expansion and persistence (57). Third-generation CAR-T cells combined multiple costimulatory domains to further augment cytotoxic activity and durability, though they were associated with higher toxicity and accelerated T-cell exhaustion (58). Fourth-generation CAR-T cells introduced additional functional modules, such as inducible switches, suicide genes, or elements to enhance T-cell activity, which mitigated adverse events (50). Fifth-generation CAR-T cells incorporated truncated cytokine receptor motifs capable of engaging STAT3/5 signaling, providing superior immune activation and persistence (59, 60).

Despite the remarkable clinical efficacy of chimeric antigen receptor T-cell (CAR-T) therapy in patients with relapsed or refractory diffuse large B-cell lymphoma (R/R DLBCL), a subset of patients still develop primary or acquired resistance, which ultimately limits long-term therapeutic benefit. Current evidence suggests that resistance to CAR-T therapy is mainly attributable to several mechanisms. First, downregulation or loss of target antigens, particularly CD19 antigen escape, impairs effective tumor recognition by CAR-T cells and represents a major cause of disease relapse. Second, intrinsic CAR-T cell dysfunction, including insufficient *in vivo* persistence, premature T-cell exhaustion, or reduced proliferative capacity, can markedly attenuate antitumor activity. In addition, the immunosuppressive tumor microenvironment further restricts the sustained function and expansion of CAR-T cells (61–63). Collectively, these resistance mechanisms highlight that optimization of CAR construct design, multi-antigen targeting strategies, and combination approaches with immunomodulatory therapies may represent promising avenues to enhance the efficacy and durability of CAR-T cell therapy.

To further enhance the efficacy of CAR-T cell therapy in DLBCL and overcome primary or secondary resistance, multiple studies have begun exploring the combination of CAR-T cells with other therapeutic modalities. Currently, several CAR-T cell products have been approved for the treatment of DLBCL, marking a significant milestone in the application of cellular immunotherapy to hematologic malignancies.

#### 2.3.1 Axicabtagene ciloleucel

Axicabtagene ciloleucel (axi-cel; Kite Pharma) is an autologous anti-CD19 CAR-T cell therapy that utilizes the same CAR construct originally developed by the U.S. National Cancer Institute (64). A multicenter, open-label, single-arm phase II study published in December 2017 evaluated axi-cel in 101 patients with R/R DLBCL. The objective response rate (ORR) was 82%, with a complete response rate (CRR) of 54%. At a median follow-up of 15.4 months, 42% of patients maintained responses, and 40% remained in complete remission; the 18-month overall survival (OS) rate was 52%. Based on these findings, axi-cel received FDA approval in 2017 for the treatment of R/R DLBCL in patients who failed two or more prior systemic therapies (61).

ZUMA-7 was a pivotal global, multicenter, randomized phase III trial that evaluated axi-cel as second-line therapy in patients with R/R LBCL who experienced relapse or refractory disease within 12 months after first-line chemoimmunotherapy. The results demonstrated that axi-cel significantly improved event-free survival (EFS) compared with standard of care (SOC), with a median EFS of 8.3 months versus 2.0 months. Axi-cel also achieved higher complete response rates and more durable disease control. In addition, axi-cel showed clinically meaningful benefits in patient-reported outcomes (PROs), with faster recovery and more sustained improvements in health-related quality of life (65).

The ZUMA-11 trial evaluated the safety and efficacy of axi-cel combined with the 4-1BB agonist antibody utomilumab as a treatment regimen. Approximately 60% of these patients had previously demonstrated no response or ultimately relapsed following prior therapies. Results demonstrated superior therapeutic outcomes for the combination regimen compared to axi-cel monotherapy, achieving an ORR of 75% and CR of 58%, alongside more stable disease control. Furthermore, the combination regimen demonstrated manageable safety, with no dose-limiting toxicities (DLTs) observed. No ≥Grade 3 cytokine release syndrome or neurological events occurred, while key cytokines including IL-2, IFN-γ, and IL-10 exhibited dose-dependent upregulation. This provides a novel direction for enhancing the efficacy of immunotherapy in LBCL (66).

Another real-world analysis based on the French DESCAR-T registry study compared the efficacy and safety of axi-cel with another product, tisagenlecleucel (tisa-cel), following propensity score matching (PSM). The results demonstrated a more pronounced clinical benefit for axi-cel: post-matching, the ORR in the axi-cel group reached 80.4% and the CRR was 60.3%, significantly higher than the 66.0% and 42.1% observed in the tisa-cel group. At a median follow-up of 11.7 months, the 1-year PFS rate in the axi-cel group was 46.6% and the 1-year OS rate was 63.5%, both superior to the 33.2% and 48.8% rates observed in the tisa-cel group. These real-world data further validate the therapeutic value of axi-cel in relapsed/refractory DLBCL, providing additional evidence for clinical decision-making (67).

#### 2.3.2 Tisagenlecleucel

Tisagenlecleucel (tisa-cel) is a CD19-directed CAR-T cell therapy that was approved in May 2018. In a phase II international, multicenter, open-label trial conducted in 2021, 93 patients with DLBCL refractory to multiple prior lines of therapy were enrolled for efficacy evaluation. The overall response rate (ORR) was 52%, with a complete response (CR) rate of 40% and partial response (PR) rate of 12%. Among patients achieving CR or PR at 3 months, the estimated 12-month progression-free survival (PFS) rate was 83%. Grade 3–4 treatment-related adverse events (AEs) included cytokine release syndrome (CRS; 22%) and neurotoxicity (12%), with no treatment-related deaths reported. These findings indicate that tisagenlecleucel provides durable responses with manageable toxicity in heavily pretreated DLBCL (68).

The BELINDA trial (NCT03570892), published in February 2022, was an international, multicenter, randomized, open-label phase III study that enrolled a total of 322 patients, who were randomly assigned to receive either tisagenlecleucel (n = 162) or standard of care (SOC; n = 160). The results showed that the median EFS was 3.0 months in both groups, with no significant improvement observed in the tisagenlecleucel group compared with the SOC group. The ORR was 46.3% in the tisagenlecleucel group and 42.5% in the SOC group, with no statistically significant difference between the two arms. These findings indicate that, in the second-line treatment of aggressive B-cell lymphoma after failure of first-line therapy, tisagenlecleucel did not demonstrate superiority over standard salvage therapy, and further studies are required to better define the patient populations most likely to benefit from this approach (69).

A single-center, retrospective real-world study from Korea including 96 patients treated with tisa-cel reported an ORR of 71.9% at 1 month, which declined to 55.1% at 3 months. The median PFS was 4.5 months, with a 1-year PFS rate of 33.3%, while the median OS was 13.9 months and the 1-year OS rate was 55.2%. In terms of safety, CRS occurred in 75% of patients (grade ≥3, 14.6%), and ICANS was observed in 22.9% (grade ≥3, 7.3%). These data suggest that, in a real-world setting, tisa-cel retains clinically meaningful activity with an overall manageable toxicity profile; however, response durability and the risk of early progression require careful consideration in relation to disease burden, response to bridging therapy, and other baseline clinical factors (70).

#### 2.3.3 Lisocabtagene maraleucel

Lisocabtagene maraleucel (liso-cel) is a defined-composition CD19-targeted CAR-T cell product incorporating a 4-1BB costimulatory domain, manufactured at a fixed ratio of CD8+ and CD4+ CAR+ T cells (16). Based on the results of the TRANSCEND NHL 001 trial, liso-cel received FDA approval in February 2021 for the treatment of large B-cell lymphoma (71).

In a study conducted by Jeremy S. Abramson et al., 270 patients with relapsed/refractory (R/R) DLBCL after ≥ 2 prior lines of therapy were treated with liso-cel, including 7 patients with secondary central nervous system (CNS) involvement. Among evaluable patients, the overall response rate (ORR) was 73% (187/257) and the complete response rate (CRR) was 53% (136/257), demonstrating that liso-cel induces durable disease remissions in this heavily pretreated population (72).

A global phase III randomized controlled trial compared liso-cel with standard-of-care (SOC; high-dose chemotherapy followed by autologous stem cell transplantation [ASCT]) as second-line therapy in LBCL. A total of 184 patients were randomized, with DLBCL comprising 66% and 63% of the liso-cel and SOC cohorts, respectively. At a median follow-up of 17.5 months, the liso-cel group achieved an ORR of 87% and a CRR of 74%, with median progression-free survival (PFS) and event-free survival (EFS) not reached. By contrast, the SOC group demonstrated an ORR of 49% and a CRR of 43%, with median PFS and EFS of 6.2 months and 2.4 months, respectively. These results establish the superiority of liso-cel over SOC in terms of ORR, CRR, PFS, and EFS, supporting earlier use of liso-cel to prevent progression to more refractory disease and improve survival outcomes in patients with primary refractory or early-relapsed LBCL (73).

A retrospective real-world analysis from a U.S. cell therapy consortium across seven centers summarized the use of commercial liso-cel in routine clinical practice. A total of 101 patients underwent infusion, with a median age of 71 years, a high comorbidity burden, and a subset of patients who would not have met the TRANSCEND eligibility criteria. At day 90 post infusion, the ORR was 66%, including a CR rate of 60%. With a median follow-up of 15.5 months, the 12-month PFS and OS rates were 55% and 68%, respectively. Regarding safety, CRS of any grade occurred in 49% of patients (grade ≥3, 3%), and ICANS of any grade was observed in 26% (grade ≥3, 10%). These findings indicate that, in an older and more comorbid real-world population, the effectiveness and safety profile of liso-cel are broadly consistent with those reported in pivotal trials, although standardized monitoring and supportive management for CRS and ICANS remain essential (74).

#### 2.3.4 Relmacabtagene autoleucel

Relmacabtagene autoleucel (relma-cel) is a second-generation CD19-targeted CAR-T cell product manufactured in China. In a multicenter, single-arm, phase 2 clinical trial, 58 patients with relapsed or refractory (R/R) DLBCL who had received ≥2 prior lines of therapy were evaluated for efficacy. The objective response rate (ORR) was 77.6% and the complete response rate (CRR) was 51.7%. The 12-month progression-free survival (PFS) and overall survival (OS) rates were 69.2% and 76.8%, respectively. Despite CAR-T cell levels falling below the limit of quantification in 41 patients (69.5%), nearly 40% of patients maintained sustained responses. This demonstrates significant efficacy and a favorable safety profile for relma-cel treatment (75). Based on these results, relma-cel received approval from the China National Medical Products Administration (NMPA) in June 2021 for the treatment of adult patients with R/R DLBCL following two or more prior lines of systemic therapy.

A single-center, retrospective, real-world cohort study in China included 33 patients with R/R large B-cell lymphoma treated with commercial CD19 CAR-T (relma-cel 20, axi-cel 13). Overall, the 3-month CR rate was 76.7%, 1-year OS 72.3%, and 1-year PFS 71.2%. In the relma-cel group, the 3-month CR rate was 61.1%. CRS occurred in 31.6% grade 0, 57.9% grade 1, and 10.5% grade 2, with no grade ≥3 events; ICANS occurred in 5.3% grade 1, with 94.7% unaffected and no grade ≥3 events. These results suggest that relma-cel achieves high remission with a low incidence of severe CRS/ICANS in the Chinese real-world setting, but further validation in larger, multicenter studies is warranted (76).

The specific clinical efficacy and safety data of the aforementioned CAR-T cell therapies in DLBCL are summarized in **Table 1**.

**Table 1:** Clinical efficacy and safety data of CAR-T cell therapy.

CAR-T product	Target	Clinical trial ID	Number	ORR	CRR	Median PFS	Median OS
Axicabtagene Ciloleucel	CD19	NCT04608487	101(DLBCL, 101, 100%)	82%	54%	5.8months	NR
Axicabtagene Ciloleucel	CD19	NCT03391466	359(DLBCL, 359, 100%)	NR	NR	8.3months	NR
Axicabtagene Ciloleucel	CD19	NCT03704298	12(DLBCL, 12, 100%)	75%	58%	NR	NR
Tisagenlecleucel	CD19	NCT02445248	93(DLBCL, 93, 100%)	52%	40%	NR	12months
Tisagenlecleucel	CD19	NCT03570892	162(DLBCL, 162, 100%)	46.3%	28.4%	NR	NR
liso-cel	CD19	NCT02631044	270(DLBCL, 216, 80%)	73%	53%	6.8months	27.3months
Lisocabtagene maraleucel	CD19	NCT03575351	184(DLBCL, 118, 64%)	87%	74%	NR	NR
Relmacabtagene Autoleucel	CD19	NCT04089215	58(DLBCL, 58, 100%)	77.6%	51.7%	7months	NR

### 2.4 Antibody–drug conjugates

Antibody–drug conjugates (ADCs) have recently emerged as a highly promising therapeutic modality for diffuse large B-cell lymphoma (DLBCL). An ADC consists of three principal structural components: an antibody that selectively recognizes a tumor-associated antigen capable of internalization; a cytotoxic payload that exerts cell-killing activity upon intracellular release; and a linker that covalently connects the antibody to the payload (2, 77). These components are critical determinants of ADC design, and the therapeutic efficacy of each ADC largely depends on variations in these three basic elements (78).

The antibody moiety typically targets cell-surface antigens that are selectively expressed or overexpressed on malignant cells relative to normal tissues (79). ADCs achieve tumor cell killing by delivering potent cytotoxic payloads in a targeted manner, while preserving the favorable pharmacokinetic and biodistribution properties of immunoglobulins and, in some cases, retaining their intrinsic biological or immunomodulatory activity. The choice of antibody, linker chemistry, cytotoxic payload, and the optimal drug-to-antibody ratio (DAR) is generally guided by empirical evidence, with the primary objective of maximizing the therapeutic index (80–83).

By combining the target selectivity of monoclonal antibodies (mAbs) with the cytotoxic potency of chemotherapeutic agents, ADCs represent a potent treatment modality for various cancers, including DLBCL (84). Several ADCs targeting distinct antigens and employing diverse payloads have been developed and are under clinical investigation for DLBCL, with multiple studies demonstrating encouraging efficacy and manageable safety profiles.

#### 2.4.1 CD79b ADCs (polatuzumab vedotin)

CD79b is a component of the B-cell receptor (BCR) complex and is widely expressed on the surface of mature B cells, including DLBCL cells, making it a common target for anti-tumor agents (9). Polatuzumab vedotin (Pola) is a second-generation antibody-drug conjugate (ADC) consisting of a humanized monoclonal antibody (mAb) targeting CD79b linked to monomethyl auristatin E (MMAE). The MMAE payload is conjugated via a protease-cleavable peptide linker to cysteine residues on the antibody, enabling targeted delivery of the cytotoxic agent into malignant B cells (85).

An international, multicenter, open-label phase Ib/II trial compared the efficacy of Pola combined with bendamustine plus rituximab (Pola-BR) versus BR alone in transplant-ineligible patients with R/R DLBCL. The results demonstrated that although grade 3–4 neutropenia, anemia, and thrombocytopenia were more frequent in the Pola-BR group, the CR rate was 40%, compared to 17.5% with BR monotherapy. Both PFS and OS were significantly improved with Pola-BR, leading to FDA approval in 2019 for this combination in patients with R/R DLBCL who had received at least two prior therapies (86).

POLARIX is an international, multicenter, randomized, double-blind phase III trial (NCT03274492) comparing pola-R-CHP with R-CHOP in previously untreated intermediate- to high-risk DLBCL. After a median follow-up of 28.2 months among 879 patients, 2-year PFS was 76.7% vs 70.2%, with longer duration of response in complete remission, while 2-year OS was similar (88.7% vs 88.6%). Adverse event type and incidence were comparable. These results indicate that pola-R-CHP is the first first-line regimen in nearly 20 years to surpass R-CHOP, providing a new standard of care for intermediate- to high-risk patients (87).

To determine if Pola-R-CHP significantly improves progression-free survival (PFS) compared to R-CHOP in previously untreated DLBCL patients and identify the optimal patient population, a study enrolled 30 treatment-naïve elderly DLBCL patients receiving Pola-R-CHP and compared them to 100 matched controls treated with R-CHOP or R-THP-COP. At the end of treatment, the Pola-R-CHP group achieved an ORR of 93.3% and a CR rate of 86.7%, compared to a CR rate of 76.0% in the control group. The 6-month PFS rate was 93.3% for Pola-R-CHP, significantly higher than the 80.0% observed in the control group. This demonstrates the promising efficacy of Pola-R-CHP compared to R-CHOP-based regimens (88).

The phase Ib/II trial and subsequent analyses demonstrate the favorable clinical outcomes of Pola-BR in R/R DLBCL. Building on these findings, researchers have explored the potential of Pola in combination with other regimens. An open-label, multicenter study investigated whether adding venetoclax to Pola and rituximab (Pola-Ven-R) could enhance anti-tumor responses. The investigator-assessed CR rate was 31%, with a median PFS of 4.4 months and median OS of 11 months. The safety profile of Pola-Ven-R was consistent with the known safety of the individual agents, demonstrating promising activity (89).

#### 2.4.2 CD19 ADCs(loncastuximab tesirine)

Loncastuximab tesirine (LT) is an antibody-drug conjugate (ADC) composed of a humanized antibody targeting CD19 conjugated to tesirine. The tesirine payload consists of a valine-alanine linker and a warhead, SG3199, which is a pyrrolobenzodiazepine (PBD) dimer (90, 91).

In a multicenter, open-label, single-arm phase II trial, 184 patients with relapsed or refractory (R/R) DLBCL following multiple prior systemic therapies were screened, and 145 patients (79%) received at least one dose of loncastuximab tesirine. The overall response rate (ORR) was 48.3% (70/145), with 35 complete responses (CRs) and 35 partial responses (PRs). The most common grade ≥3 adverse event (AE) was neutropenia, occurring in 37 patients. These findings confirmed the single-agent antitumor activity of loncastuximab tesirine, establishing it as a novel therapeutic option for DLBCL (92).

Additionally, real-world studies have evaluated LT in patients who relapsed after CAR-T therapy. In a cohort of 118 patients who received LT monotherapy as third-line (3L) or fourth-line (4L) treatment following second-line (2L) or third-line (3L) CAR-T therapy, 95 patients who received 2L CAR-T followed by 3L LT achieved an ORR of 73% with a CR rate of 34%. Among 23 patients who received 3L CAR-T followed by 4L LT, the ORR was 78% and the CR rate was 17%. These results suggest that LT monotherapy is an effective therapeutic option for 3L and 4L treatment of R/R DLBCL (93).

Additionally, clinical studies investigating LT combined with other immunochemotherapies are ongoing, such as a phase III trial combining it with rituximab plus gemcitabine and oxaliplatin (R-GemOx) in patients with R/R DLBCL (NCT04384484) (94).

#### 2.4.3 CD22 ADCs(inotuzumab ozogamicin)

Inotuzumab ozogamicin (CMC-544) is an antibody-targeted chemotherapeutic agent composed of a humanized anti-CD22 antibody conjugated to the potent cytotoxic agent calicheamicin.

A randomized, open-label phase III trial evaluated the efficacy of inotuzumab ozogamicin combined with rituximab (R-InO) versus investigator’s choice of rituximab-based chemotherapy (IC: R-B or R-G) in 338 patients. Patients were randomized to R-InO (n = 166) or IC (n = 172). After a median follow-up of 14.9–15.9 months, no significant differences were observed between the two groups in OS (both 9.5 months), PFS (3.7 vs 3.5 months), or ORR (41% vs 44%). However, the R-InO group showed a longer duration of response (DOR, 11.6 vs 6.9 months), and patients with high baseline CD22 expression appeared to benefit more. These results indicate that R-InO has antitumor activity in patients with limited treatment options but does not surpass IC, and its role in specific patient subgroups warrants further investigation (95).

#### 2.4.4 CD30 ADCs (brentuximab vedotin)

Brentuximab vedotin (BV) is a CD30-directed monoclonal antibody conjugated to monomethyl auristatin E (MMAE), a potent inhibitor of microtubule polymerization, which selectively targets and eliminates CD30-expressing lymphoma cells (96).

A phase II study investigated the efficacy of Brentuximab vedotin combined with R-CHOP as first-line therapy for patients with intermediate-high-risk DLBCL. Fifty-one patients were enrolled. The overall PET-negative complete response (CR) rate was 69%. The CR rate was 76% in CD30-positive patients and 63% in CD30-negative patients (97). Importantly, the randomized, double-blind, placebo-controlled, multicenter phase 3 ECHELON-3 trial evaluated brentuximab vedotin plus lenalidomide plus rituximab (BV + Len + R) versus placebo + Len + R in heavily pretreated relapsed/refractory DLBCL (≥2 prior lines) who were ineligible for stem-cell transplant or CAR-T cell therapy. With a median follow-up of 16.4 months, BV + Len + R significantly improved overall survival (OS) (median OS 13.8 vs 8.5 months), and also improved PFS (4.2 vs 2.6 months) and ORR/CR rates (ORR 64% vs 42%; CR 40% vs 19%). Treatment-emergent adverse events occurred in 97% of patients in both arms; the most common events included neutropenia, thrombocytopenia, diarrhea, and anemia. Peripheral neuropathy was more frequent in the BV + Len + R arm (any grade 31% vs 24%; grade 3: 6% vs 2%), highlighting the need for careful toxicity monitoring.p (98).

The specific clinical research data of the aforementioned antibody-drug conjugates (ADCs) in DLBCL are summarized in **Table 2**.

**Table 2:** Clinical Research Data of Antibody-Drug Conjugates (ADCs).

ADCs	Target	Payload	Trial ID	Phase	ORR	CRR	Median PFS
POLA	CD79b	MMAE	NCT02257567	II Phase	62.50%	40%	9.5 months
POLA	CD79b	MMAE	NCT03274492	Phase III	85.5%	78.0%	NR
POLA	CD79b	MMAE	NCT02611323	Phase Ib/II	65%	31%	4.4 months
POLA	CD79b	MMAE	N/A	Retrospective study	93.30%	86.70%	NR
Loncastuximab tesirine	CD19	SG3199	NCT03589469	II Phase	48.30%	24.10%	NR
Loncastuximab tesirine	CD19	SG319921	N/A	Real-world study	78%	17%	12 months
Inotuzumab Ozogamicin	CD22	Calicheamicin	NCT01232556	III phase	41%	13%	3.7 months
Brentuximab Vedotin	CD30	MMAE	NCT01925612	II Phase	NR	69%	NR
Brentuximab Vedotin	CD30	MMAE	NCT04404283	III Phase	64%	40%	4.2 months

### 2.5 Bispecific antibodies

The concept of bispecific antibodies (bsAbs) was first proposed three decades ago, when Morrison and colleagues engineered the first genuine bsAb by fusing a flexible linker peptide to the C-terminus of the IgG heavy chain (99). Bispecific antibodies exert their effects by simultaneously binding to two distinct antigenic epitopes, thereby offering enhanced and more precise immunologic targeting compared with native monoclonal antibodies (100). Common bsAbs investigated for the treatment of DLBCL include CD19/CD3 and CD20/CD3 bispecific antibodies.

#### 2.5.1 CD19/CD3

Blinatumomab is a CD19 × CD3 bispecific T-cell engager (BiTE) antibody that transiently links CD19-positive B cells with CD3-positive T cells, thereby inducing T-cell–mediated B-cell lysis and T-cell proliferation. It is the first immunotherapy approved for adult relapsed/refractory (R/R) B-cell acute lymphoblastic leukemia (B-ALL) due to its unique mechanism of action, manageable toxicity profile, and high response rates. The U.S. Food and Drug Administration (FDA) approved blinatumomab for R/R B-ALL treatment in 2014 (101, 102).

Multiple clinical trials have explored the application of blinatumomab in DLBCL treatment. A phase II study evaluating stepwise dosing in R/R DLBCL enrolled 21 evaluable patients, with an overall response rate (ORR) of 43% and a complete remission (CR) rate of 19% after one treatment cycle. The most common adverse events associated with stepwise dosing included tremor (48%), pyrexia (44%), fatigue (26%), and edema (26%). Although blinatumomab monotherapy demonstrated efficacy in R/R DLBCL, optimal dosing regimens require further investigation (103).

In an open-label, multicenter phase II trial, blinatumomab was administered to patients previously treated with rituximab-containing chemotherapy. Among 47 patients, an ORR of 89% was observed; with a median follow-up of 8.6 months, 93% of patients remained alive, underscoring blinatumomab’s therapeutic potential in R/R DLBCL (104).

Another study investigating blinatumomab combined with lenalidomide enrolled 18 patients, reporting an ORR of 56% in the entire cohort and 83% in the combination group, with a CR rate of 50%. Median progression-free survival (PFS) was 3.8 months for all patients and extended to 8.3 months in the combination arm, demonstrating the combination’s favorable safety and durability (105).

#### 2.5.2 CD20/CD3

##### 2.5.2.1 Mosunetuzumab

Mosunetuzumab is a fully humanized IgG1 antibody targeting CD20 and CD3. A first-in-human trial assessed the safety, tolerability, and efficacy of Mosunetuzumab in patients with R/R B-cell NHL and established the recommended phase II dose. The most common adverse events occurring in ≥20% of patients included neutropenia (28.4%), cytokine release syndrome (27.4%), hypophosphatemia (23.4%), fatigue (22.8%), and diarrhea (21.8%) (106).

Based on the phase I dose-escalation experience, a single-arm expansion cohort of the open-label phase 1/2 study (NCT02500407) further evaluated fixed-duration intravenous mosunetuzumab in patients with relapsed/refractory (R/R) DLBCL (including transformed follicular lymphoma) who had received ≥2 prior lines of therapy and were ineligible for ASCT. Mosunetuzumab was administered with cycle 1 step-up dosing for CRS mitigation. Among 88 enrolled patients, the ORR and CR rates were 42.0% and 23.9%, respectively; the median time to first response was 1.4 months and the median PFS was 3.2 months. CRS occurred in 26.1% of patients and was predominantly grade 1–2 and mainly confined to cycle 1; grade 3 CRS was uncommon (2.3%) with no grade 4–5 events. This indicated that its overall therapeutic efficacy is safe and controllable (107).

In a study evaluating Mosunetuzumab combined with CHOP (M-CHOP) for non-Hodgkin lymphoma (NHL), 43 patients were treated. Among them, 7 patients with relapsed/refractory (R/R) NHL achieved an overall response rate (ORR) of 86% and a complete remission (CR) rate of 71%; 27 newly diagnosed DLBCL patients showed an ORR of 96% and a CR rate of 85%. These results suggest that Mosunetuzumab combined with CHOP holds promising therapeutic potential for R/R NHL and DLBCL (108).

Mosunetuzumab can be administered intravenously or subcutaneously. A phase I/Ib, open-label, multicenter dose-escalation and expansion study evaluated subcutaneous Mosunetuzumab in patients with B-cell lymphomas, including 22 evaluable patients, of whom 10 had DLBCL. The ORR and CR rates for indolent NHL were 86% (6/7) and 29% (2/7), respectively, while for aggressive NHL, the ORR and CR rates were 60% (9/15) and 20% (3/15), respectively (109).

##### 2.5.2.2 Glofitamab

Glofitamab is a bispecific antibody targeting CD20 and CD3, distinguished by its structure comprising two CD20-binding domains and one CD3-binding domain, which enhances its binding affinity to B cells.

A multicenter, phase 1/2 dose-escalation and expansion clinical trial, designed to evaluate the efficacy of glofitamab, enrolled 38 patients with relapsed or refractory non-Hodgkin lymphoma (NHL), including 12 patients with relapsed/refractory diffuse large B-cell lymphoma (R/R DLBCL). All these patients uniformly received pre-treatment with 1000 mg obinutuzumab 7 days prior to the first administration of glofitamab. After a median follow-up of 2.8 months, the overall response rate (ORR) was 62.5% and the complete metabolic response (CMR) was 40.6% among 32 evaluable patients. This study indicated that the treatment regimen of glofitamab in combination with obinutuzumab could provide a new effective option for NHL patients who had failed multiple lines of treatment, especially those refractory to CD20-targeted therapy. However, adverse events such as fever (31.6%) still occurred in the treated patients. More patients will be enrolled subsequently to further verify the long-term efficacy and safety of this regimen (110).

A phase II study enrolled 155 patients with relapsed/refractory (R/R) DLBCL who had previously received at least two lines of therapy. Patients underwent obinutuzumab pretreatment followed by fixed-duration monotherapy with Glofitamab for 12 cycles. After a median follow-up of 12.6 months, the complete remission (CR) rate was 39%, and the overall response rate (ORR) was 52%. The 12-month progression-free survival (PFS) and overall survival (OS) rates were 37% and 50%, respectively. Treatment discontinuation due to adverse events occurred in 9% of patients, while grade 3 or higher adverse events were reported in 62%. These findings indicate that fixed-duration Glofitamab therapy can enhance treatment efficacy but requires careful monitoring for potential toxicities (111).

##### 2.5.2.3 Epcoritamab

Epcoritamab, a subcutaneously administered CD3×CD20 bispecific antibody, is used to treat various R/R LBCL (including DLBCL) patients and is currently a preferred regimen for third-line and subsequent therapy in DLBCL (112).

The EPCORE NHL-1 study evaluated epcoritamab in patients with relapsed or refractory LBCL after ≥2 prior therapies. At a median follow-up of 25.1 months, the ORR was 63.1%, with a CR rate of 40.1%. The 24-month PFS and OS rates were 27.8% and 44.6%, respectively, and 64.2% of patients achieving CR maintained their response through 24 months. CRS occurred in 51.0% of patients, predominantly grade 1–2. These results provided key supportive evidence for FDA approval (112).

Joshua D. Brody et al. reported results from the phase 1b/2 EPCORE NHL-2 trial evaluating epcoritamab plus GemOx in transplant-ineligible R/R DLBCL patients (N = 103). The objective response rate (ORR) and complete response (CR) rate were 85% and 61%, respectively. The median duration of CR and overall survival (OS) were 23.6 months and 21.6 months. Common treatment-emergent adverse events included cytopenias and cytokine release syndrome (CRS). These results demonstrate that epcoritamab plus GemOx induces deep and durable responses, providing a highly effective and well-tolerated novel option for transplant-ineligible R/R DLBCL patients (113).

The specific clinical research efficacy data of the aforementioned bispecific antibodies in DLBCL are summarized in **Table 3**.

**Table 3:** Clinical research efficacy data of bispecific antibodies.

Trial ID	Bispecific agent	Target combination	Indication	Phase	Patient	ORR	CRR
NCT01741792	Blinatumomab	CD19/CD3	R/R DLBCL	Phase II	21	43%	19%
NCT03023878	Blinatumomab	CD19/CD3	R/R DLBCL	Phase II	47	89%	NR
NCT02568553	Blinatumomab	CD19/CD3	R/R DLBCL	Phase I	18	83%	50%
NCT03677141	Mosunetuzumab	CD20/CD3	R/R NHL and DLBCL	Phase II	43	96%	85%
NCT02500407	Mosunetuzumab	CD20/CD3	R/R DLBCL (≥2 lines)	Phase II	88	42%	24%
NCT03075696	GLOfitamab	CD20/CD3	R/R DLBCL (≥2 lines of therapy)	Phase II	155	39%	52%
NCT03625037	Epcoritamab	CD20/CD3	R/R LBCL	Phase I/II	157	63%	40%
NCT04663347	Epcoritamab	CD20/CD3	R/R DLBCL (≥3 lines of therapy)	Ib/Phase II	103	85%	61%

### 2.6 Information on novel drug therapy

In addition to established immunotherapeutic modalities, several novel agents and technologies are currently under clinical investigation and may further expand the treatment landscape of DLBCL.

The efficacy and safety of magrolimab combined with rituximab (M+R) and further combined with GemOx (M+R-GemOx) were evaluated in an open-label, multicenter phase Ib/II trial (NCT02953509) in patients with relapsed or refractory DLBCL. A total of 132 patients were enrolled, with 99 receiving M+R and 33 receiving M+R-GemOx. In the M+R group, the ORR was 24%, CR rate 12%, median duration of response (DOR) 9.3 months, median PFS 1.8 months, and median OS 9.2 months. In the M+R-GemOx group, the ORR was 52%, CR rate 39%, 12-month DOR rate 66.6%, median PFS 3.9 months, and median OS was not reached. These results indicate that combining magrolimab with GemOx achieves higher response rates, but further studies are needed to define the optimal patient population due to the lack of randomized controls (114).

## 3 Conclusion

Although frontline R-CHOP has significantly improved survival outcomes in patients with diffuse large B-cell lymphoma (DLBCL), approximately 30–40% of patients still experience relapse or refractory disease due to therapeutic resistance. In recent years, advances in immunotherapy have led to meaningful progress in the management of relapsed/refractory (R/R) DLBCL. Chimeric antigen receptor T-cell (CAR-T) therapies, including axicabtagene ciloleucel and tisagenlecleucel, have demonstrated the potential to induce durable remissions in a subset of patients, although careful toxicity monitoring remains essential. Antibody–drug conjugates (ADCs), such as polatuzumab vedotin and loncastuximab tesirine, provide targeted cytotoxic delivery and have expanded treatment options for patients with refractory disease. In addition, bispecific antibodies, exemplified by mosunetuzumab, offer an “off-the-shelf” immunotherapeutic approach by redirecting T cells against malignant B cells.

Despite these advances, several challenges remain, including the biological complexity of resistance mechanisms, heterogeneity in treatment response, and the absence of standardized molecular stratification across clinical settings. Future efforts should focus on integrating multi-omics data to refine disease classification, optimizing rational combination strategies with acceptable toxicity profiles (such as CAR-T–based combinations), and further incorporating clinically applicable biomarkers to guide patient selection and therapeutic sequencing. Collectively, these approaches may facilitate more precise and individualized immunotherapeutic strategies for patients with DLBCL.
